# Fluorescent Labeling of Polymannuronic Acid and Its Distribution in Mice by Tail Vein Injection

**DOI:** 10.3390/md20050289

**Published:** 2022-04-25

**Authors:** Shuliang Song, Qiang Wei, Ke Wang, Qiong Yang, Yu Wang, Aiguo Ji, Guanjun Chen

**Affiliations:** 1 Marine College, Shandong University, Weihai 264209, China; songshuliang@sdu.edu.cn (S.S.); wq18086420598@163.com (Q.W.); wk1308866506@163.com (K.W.); yangqiong1237@163.com (Q.Y.); wy392191187@163.com (Y.W.); 2 Shandong University Weihai Research Institute of Industrial Technology, Weihai 264209, China

**Keywords:** polymannuronic acid, FITC, tissue distribution, pharmacokinetics, plasma concentration

## Abstract

Polymannuronic acid (PM) possesses more pharmacological activities than sodium alginate, but there have been few studies on its absorption mechanism, tissue distribution, and pharmacokinetics. Studies of pharmacokinetics and tissue distribution are necessary to elucidate the pharmacological effects of PM. Thus, we used fluorescein isothiocyanate (FITC) to produce fluorescently labeled PM (FITC-PM) and detected the distribution and pharmacokinetics of PM in vivo via tail vein injection. The results demonstrate that the FITC-PM showed high stability in different pH solutions. After the tail vein injection, FITC-PM tended to be distributed in the kidney, followed by the liver and in the heart, spleen, and lungs at lower concentrations. Pharmacokinetic analysis showed that the elimination rate constant of FITC-PM was 0.24, the half-life time was 2.85 h, the peak concentration was 235.17 μg/mL, the area under the curve was 631.48 μg/mL·h, the area under the curve by statistical moment was 1843.15 μg/mL·h^2^, the mean residence time was 2.92 h, and the clearance rate was 79.18 mL/h. These results indicate that FITC-PM could be used for PM distribution and pharmacokinetic studies, and the studies of pharmacokinetics and tissue distribution provided basic information that can be used to further clarify PM pharmacodynamic mechanisms.

## 1. Introduction

**Figure 1:**
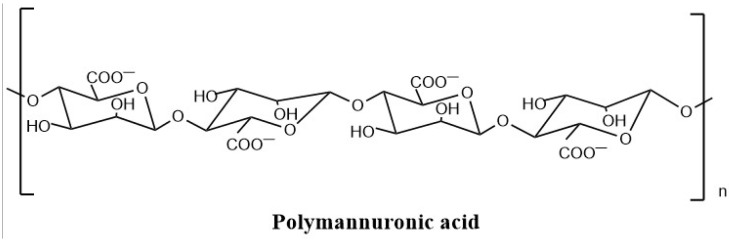
The structure of polymanuronic acid.

Sodium alginate, a linear polysaccharide extracted from brown algae cell walls, is composed of β-D-mannuronic acid (M) and α-L-guluronic acid (G) that are linked via the copolymerization of 1,4-glycosidic bonds [1]. At present, sodium alginate is widely used in various industries such as food, agriculture, textile, and medicine [2,3]. As a kind of edible macromolecular polysaccharide, sodium alginate has no significant pharmacological activity. However, some studies have shown that the oligosaccharide products obtained from sodium alginate degradation have excellent biological and pharmacological activities. Polymannuronic acid (PM) (nonsulfated polysaccharide, Figure 1), an active fragment of sodium alginate, possesses more pharmacological activities than sodium alginate itself, including anti-tumor [4], antioxidant [5], anticoagulation [6], immune regulation [7], anti-inflammatory [8], neuroprotection [9], and hypolipidemic [10], and other properties [11].

These studies mainly focus on in vitro cell activity and overall animal activity, and there have been few studies on the absorption mechanisms, tissue distribution, and pharmacokinetics of PM. Elucidating the pharmacokinetics and tissue distribution of PM is of great significance for improving the efficacy of PM, clarifying its mechanism of action, developing new pharmacological activities, guiding the dose and pathway of PM administration, and promoting the research and development of new PM drugs. Therefore, in order to preliminarily understand the pharmacokinetics and tissue distribution of PM, we plan to conduct relevant studies. Owing to a lack of chromophore and light-absorbing groups in PM, other biological components easily interfere with the detection of PM concentrations in vivo, which makes it difficult to establish an effective analytical method. In our previous study, PM was labeled with fluorescein isothiocyanate (FITC) to synthesize FITC-PM, detected the cytotoxicity by MTT, and then the PM-transport mechanism was studied using Caco-2 cell monolayer model [12]. The results of the study provided the first detailed information on the mechanisms of PM transport across intestinal epithelial cells in vitro: the PM had no measurable cytotoxicity, the transportation of PM in Caco-2 cells was a time-dependent and energy-dependent process with carrier saturation and efflux. The transport process is mediated through macropinocytosis, clathrin, and caveola (or lipid raft)-related pathways. Our group previously found that PM can be absorbed and transported by intestinal epithelial cells via endocytosis. These studies were limited in vitro experiments, and PM shows a very low oral transport rate (at 50 μg/mL, the transport rate of PM is only 1.71%), which makes it difficult to achieve an effective blood concentration [12]. There have been no studies on the distribution or pharmacokinetics of PM in vivo conducted so far. Based on the production of FITC-PM, we emphatically focused on the tissue distribution and pharmacokinetics of PM in mice to obtain information that will help with further studies concerning the pharmacodynamic mechanisms of PM and its application.

## 2. Results

### 2.1. Preparation and Purification of PM

**Figure 2:**
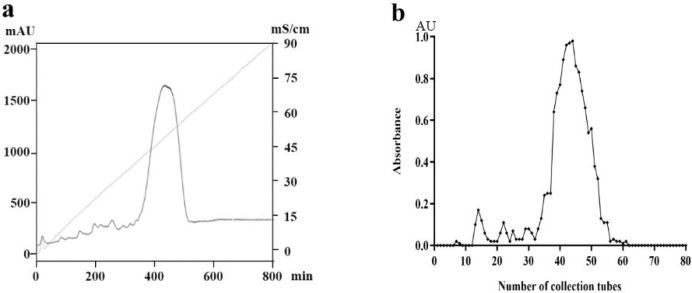
Separation and purification of PM. (**a**) Elution curve (210 nm) of PM on the Q-Sepharose ion column; (**b**) phenol–sulfuric acid method monitoring diagram of PM.

By acid degradation (0.5 M hydrochloric acid) and three pH grades, we obtained 27.5 g of crude PM from 100 g of sodium alginate, with a yield rate of 27.5%. The components of crude PM were eluted via Q-Sepharose column and detected by the phenol–sulfuric acid method to obtain the elution curve (Figure 2). The 210 nm UV elution curve (Figure 2a) showed a main elution peak corresponding to the PM component with small amounts of other components, and the detection curve of the phenol–sulfuric acid method was almost entirely consistent with the 210 nm elution curve (Figure 2b). The symmetrical elution peak indicated that the molecular weight of this component was relatively uniform. The main components were collected, desalted, lyophilized, and tested for purity by high-performance liquid chromatography.

### 2.2. Purity and Molecular Weight Determination of PM

According to the results of the HPLC chromatogram detection, the logarithm of the molecular weight of dextran (log Mw) of the standard molecular weight was used to perform regression analysis relative to the retention time (t), and the following linear regression equation was obtained:

log Mw = −0.4482t + 9.2931, R^2^ = 0.9992

**Figure 3:**
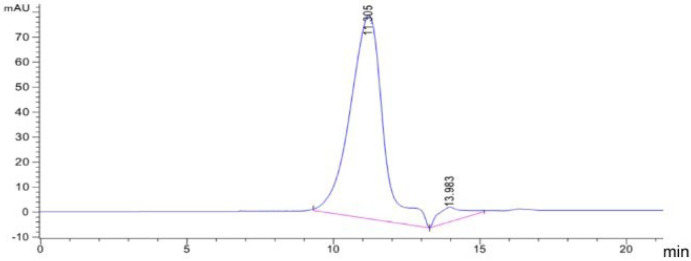
Results of high-performance liquid chromatography of PM. Elution curve (210 nm) of PM on TSK-gel G4000 PWxl type column.

The high-performance liquid chromatography analysis results for the purified PM are shown in Figure 3. The purified PM showed a single symmetrical peak, a retention time of 11.305 min, and Mw (PM) = 16.83 kDa, by calculating the molecular weight of mannuronic acid, the average degree of polymerization of PM was 95.

### 2.3. Validation of FITC-PM Labeling

**Figure 4:**
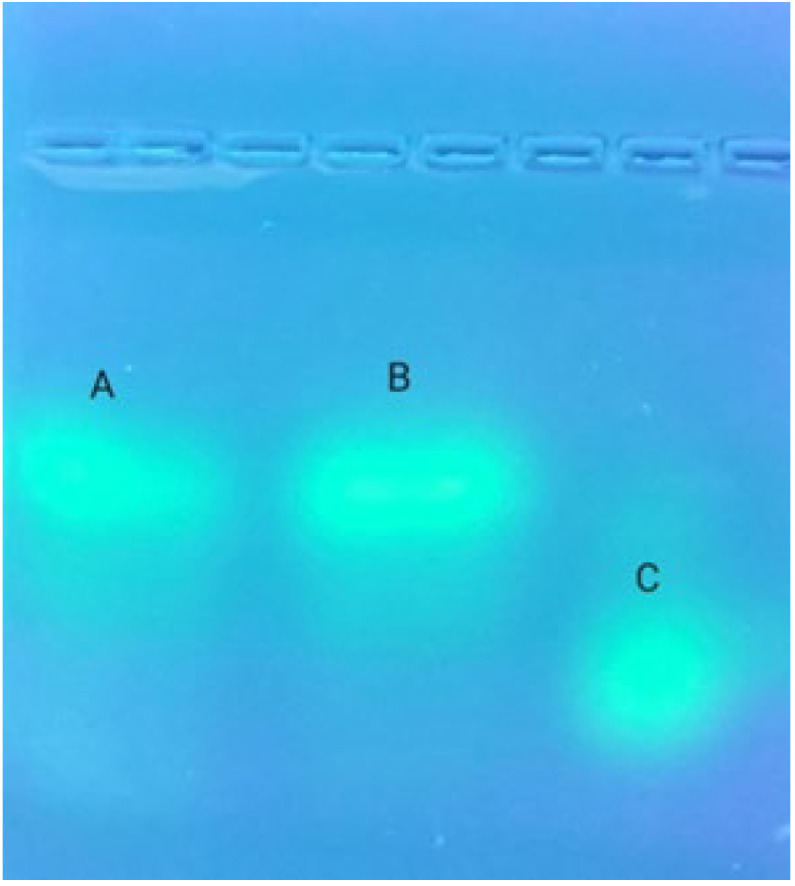
Result of FITC-PM after agarose electrophoresis. Lanes A and B, FITC-PM; lane C, FITC.

We first linked tyramine to the PM terminal, and then linked FITC to tyramine. Through this method, we obtained fluorescently labeled PM. To verify whether PM had been labeled with FITC, the FITC-PM was desalted with AKTA exporter 10 protein purification system, run through a Sephadex G-25 column, and detected by agarose electrophoresis. The results (Figure 4) showed that the free-FITC and FITC-PM were completely separated, and there was almost no free FITC in the FITC-PM fraction, which indicated that our method labeled PM with FITC and effectively removed free-FITC.

### 2.4. Stability Study of FITC-PM

**Figure 5:**
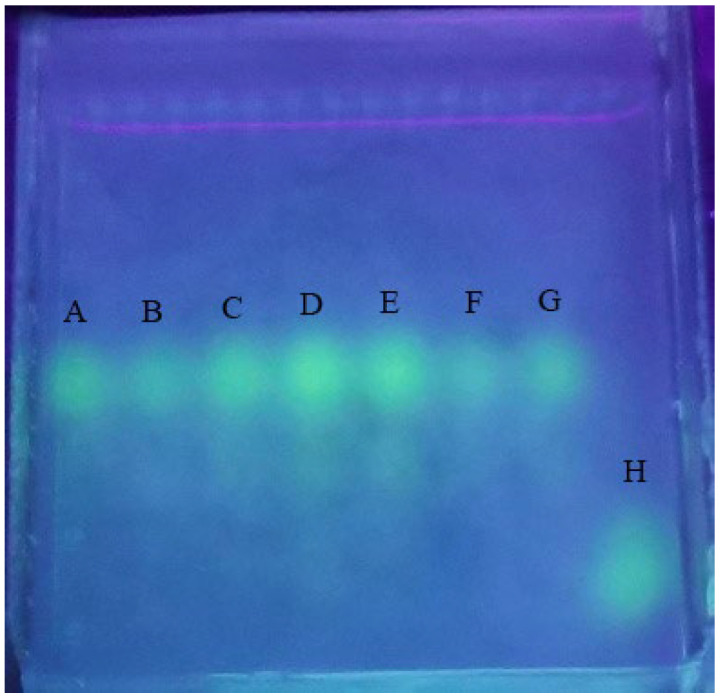
Electropherograms of FITC-PM after incubation at different pH values for 24 h. pH: Lanes A, 2; Lanes B, 4; Lanes C, 6; Lanes D, 7; Lanes E, 8; Lanes F, 10; Lanes G, 12; Lanes H, FITC.

The FITC-PM was incubated in different pH values at 37 °C for 24 h. The results (Figure 5) indicated that the molecular weight of FITC-PM was stably maintained in the pH range 2–12 for 24 h. FITC-PM is stable at different pH levels within 24 h and can be used for the detection of PM tissue distribution and pharmacokinetics research in vivo.

### 2.5. In Vivo Tissue Distribution and Pharmacokinetic Analysis of PM

#### 2.5.1. Free-FITC Interference Detection

Interference detection showed no residual free-FITC in the organ homogenate samples, which indicated that free-FITC does not affect the detection of FITC-PM in mice organ samples after the blood and tissues processing methods described in this paper.

#### 2.5.2. Standard Curve of FITC-PM in Blood and Tissues

The standard curves for FITC-PM in blood and tissues were obtained by detecting the fluorescence intensity with the cell imaging microplate detection system (Bio-Tek, Cytation 5, Norcross, GA, USA):

Standard curve of FITC-PM in heart: y = 1764x + 112446; R^2^ = 0.997

Standard curve of FITC-PM in liver: y = 748.2x + 105808; R^2^ = 0.994

Standard curve of FITC-PM in spleen: y = 1148.7x + 99459; R^2^ = 0.995

Standard curve of FITC-PM in lungs: y = 932.7x + 98000; R^2^ = 0.996

Standard curve of FITC-PM in kidney: y = 894.8x + 93641; R^2^ = 0.992

Standard curve of FITC-PM in blood: y = 1571x + 85403; R^2^ = 0.991

**Figure 6:**
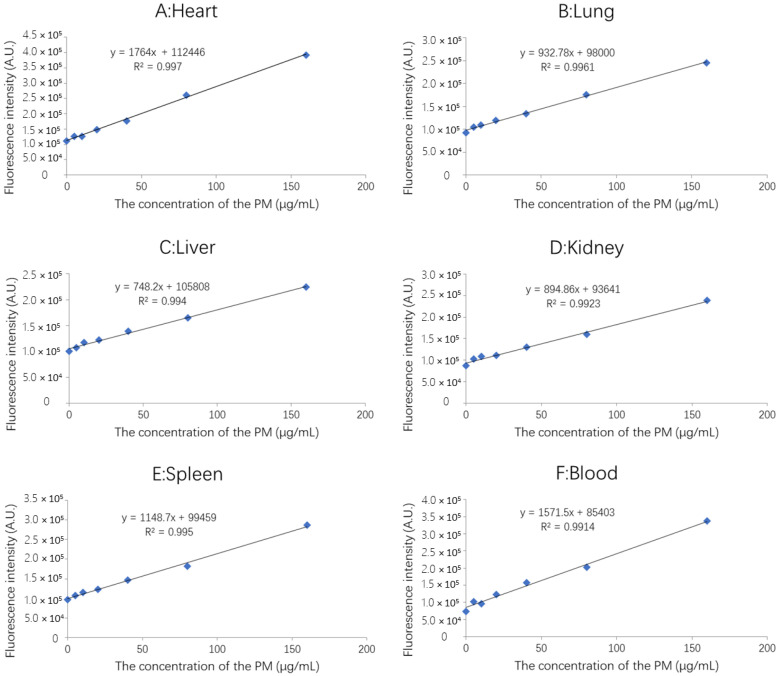
The standard curves for FITC-PM in blood and tissues. (**A**): Heart; (**B**): Lung; (**C**): Liver; (**D**): Kidney; (**E**): Spleen; (**F**): Blood.

The concentration range of all standard curves (Figure 6) was 5 to 160 μg/mL.

#### 2.5.3. Concentration and Pharmacokinetics of FITC-PM in Plasma

**Figure 7:**
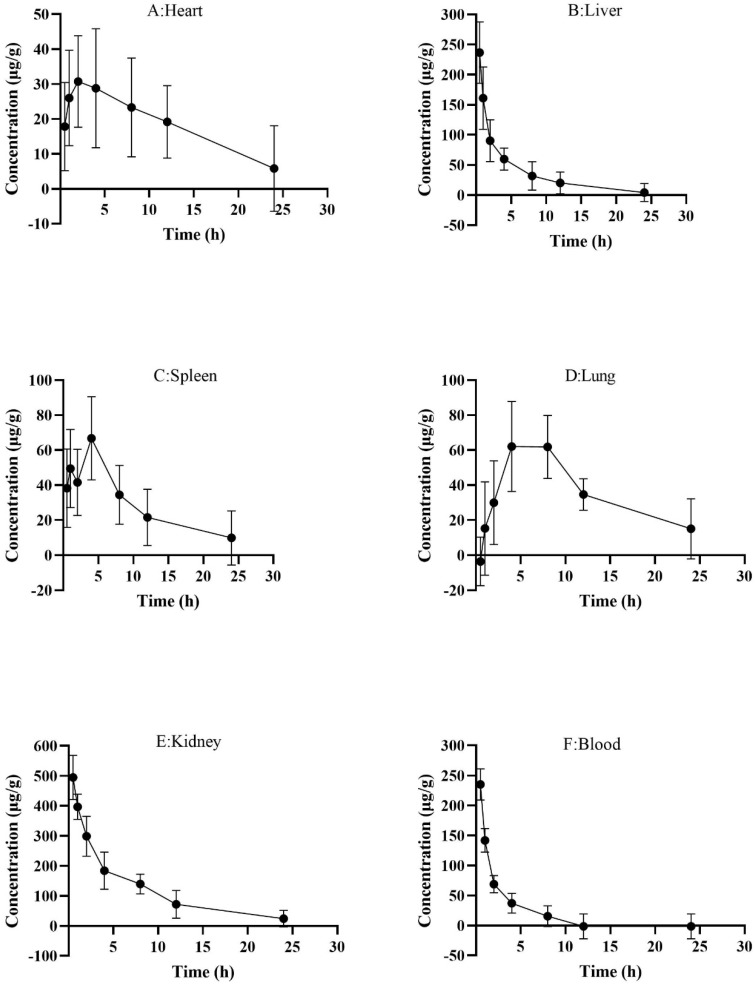
Concentrations of FITC-PM in each tissue sample at different times (mean ± SD, *n* = 6). (**A**): Heart; (**B**): Liver; (**C**): Spleen; (**D**): Lung; (**E**): Kidney; (**F**): Blood.

After FITC-PM was injected into mice tail veins, it was quickly eliminated, and the highest concentration in the blood was 235.17 μg/mL at 0.5 h, which was followed by a decreasing trend. FITC-PM was almost undetectable in the blood 8 h later (Figure 7F). However, the speed of FITC-PM elimination was quite different among mice, and there were individual differences in the response effect. The pharmacokinetic data processing software PK Solver 2.0 was used to analyze the plasma drug concentration with a noncompartmental model. The results showed that the elimination rate constant (Ke) of FITC-PM was 0.24, the half-life time was 2.85 h, the peak concentration (C_max_) was 235.17 μg/mL, the area under the curve (AUC_0–∞_) was 631.48 μg/mL·h, the area under the curve by statistical moment (AUMC_0–∞_) was 1843.15 μg/mL·h^2^, the mean residence time was 2.92 h, and the clearance rate was 79.18 mL/h (Table 1).

#### 2.5.4. Distribution of FITC-PM in Tissues

FITC-PM was primarily distributed in the kidneys, with the highest concentration being 494.50 ± 73.52 μg/g, followed by the liver, with the highest concentration of 236.67 ± 50.94 μg/g, and the heart, spleen, and lungs at lower concentrations. This indicates that uptake of PM by the kidneys and liver was high and rapid, reaching a maximum concentration by 0.5 h, followed by a decrease; FITC-PM reached a maximum concentration in the heart of 30.72 ± 13.15 μg/g at 2 h, and the maximum concentrations were reached in the spleen and lungs at 4 h, i.e., 66.74 ± 23.80 and 62.05 ± 25.72 μg/g, respectively. However, at all times, the PM concentration was highest in the kidneys. This may be because the kidneys have a strong PM-uptake capacity and because PM is mainly excreted via the kidneys. Even when PM was undetectable in the blood (Figure 7F), there were still high concentrations in the kidneys (Figure 7E).

## 3. Discussion

As a degradable oligosaccharide of sodium alginate, PM has a variety of pharmacological activities. The absorption, distribution, and pharmacokinetics of PM are important prerequisites for its further development. To study its absorption, tissue distribution, and pharmacokinetics, we must establish a sensitive PM-detection method. As an oligosaccharide, PM has no specific UV absorption spectrum and no specific color-development method has been developed, so there have been few PM-related pharmacokinetic studies [13]. In this paper, a FITC labeling method was used to fluorescently label PM, which improved the sensitivity and specificity of PM detection. On this basis, to reduce interference from other components in biological samples and further improve the specificity of detection, we used trichloroacetic acid, CTAB, and other reagents. Compared with the method of direct determination of polysaccharide content (Phenol–sulfuric acid method, Anthrone–sulfonic acid method) after extraction and purification, our method can significantly improve the specificity and sensitivity of the determination. Moreover, our method reduces and optimizes the extraction steps of biological samples, and only the total polysaccharides in the biological sample need to be extracted before the determination. In addition, we measured the stability of FITC-PM in the pH range 2–12, and the results showed that FITC-PM obtained by the covalently binding method has high stability, which can be used to study the distribution and pharmacokinetics of PM in vivo. Compared with LC–MS technology and other chromatographic analysis methods, this method requires less uniformity of molecular weight of samples, and can reflect the distribution and metabolism of PM in vivo more comprehensively. Compared with radioisotope labeling methods, the sensitivity of this method is lower, but this method does not need special and expensive instruments and radioactive reagents, only a fluorescence spectrophotometer is necessary and more suitable for ordinary laboratory use. The disadvantage of this method is that PM cannot be detected after being metabolized or degraded, and cannot reflect the degradation or metabolism of PM in vivo. In addition, the fluorescence labeling rate of PM also affects the sensitivity of PM detection, which requires further improvement to improve the labeling rate of PM.

In this study, FITC was used to label PM. Although the detection sensitivity of PM was improved, FITC may affect the tissue distribution of PM. In this study, the molecular weight of PM was determined to be 16.83 kDa by high performance gel chromatography. The molecular weight of FITC was 389.38 Da, and that of tyramine was 137.18 Da. Therefore, we hypothesized that FITC and tyramine had little effect on the tissue distribution of PM. Dong et al. also used the method of FITC labeling to study the distribution and pharmacokinetics of mulberry polysaccharides in vivo [14]. Of course, further experiments are needed to prove whether FITC affects the tissue distribution of PM, but this experiment is difficult due to the lack of a specific method to detect unlabeled PM.

After FITC-PM was injected into the mice through the tail vein, the blood concentration decreased rapidly and then slowly, and the presence of FITC-PM was almost undetectable in the blood after 8 h (Figure 7F). This indicates that PM has a nonlinear distribution, which distributes rapidly in all organs after entering the blood and can reach the target organs quickly. However, at 8–12 h, some mice still contained a certain concentration of PM in their blood, indicating that there was a large individual difference in the distribution and metabolism of PM. Therefore, attention should be paid to monitoring the blood concentration of PM during administration. The pharmacokinetic data processing software PK Solver 2.0 was used to analyze the plasma drug concentration with a noncompartmental model. The elimination rate constant of PM in mice was 0.24 and the half-life was 2.85 h, indicating that the elimination rate was relatively rapid, which may be related to the low molecular weight of PM (16.83 kDa). Therefore, PM needs to be administered at least three times a day for good efficacy. The volume of distribution was 325.46 mL, indicating that PM is widely distributed or has a large amount binding with biopolymers. This was consistent with the distribution of PM detected in multiple organs. The average residence time of PM was 2.92 h, and the clearance rate was 79.18 mL/h, indicating that the removal was relatively rapid. However, we detected that the kidney still contained a certain concentration of PM within 24 h (Figure 7E), and attention should be given to the accumulation of the concentration of PM in the kidney. Therefore, further studies of long-term toxicity are needed.

We studied the absorption characteristics of PM in vitro and obtained relatively reliable experimental results that proved the feasibility of using FITC-PM in the study of PM pharmacokinetics. We then studied the tissue distribution of PM. The results showed that, when FITC-PM was injected into mice via the tail vein, it accumulated rapidly in the kidneys and liver (the highest concentrations were reached within 0.5 h) along with the heart, spleen, and lungs. With time, the PM concentration in the blood, kidneys, and liver gradually decreased. The concentration of PM in the kidneys constantly remained the highest among all organs, which indicated that the kidney may have a stronger PM-uptake ability, and it also shows that PM is mainly excreted by the kidneys. Even when PM was undetectable in the blood, there were still high concentrations of PM in the kidneys. These results are comparable to the tissue distribution of other polysaccharides. For example, after oral administration, pumpkin polysaccharide circulates in the blood for a long time, with a mean residence time of 7.45 h, and the aggregation of pumpkin polysaccharide is highest in the liver and kidney [15]. Dong et al. also used the method of FITC labeling to study the distribution and pharmacokinetics of mulberry polysaccharides in vivo. The results showed that mulberry polysaccharides were mainly distributed in the liver and kidney and were eliminated slowly [14]. Pozharitskaya et al. showed that the distribution of fucoidan in the tissues of rats after intragastric administration was more obvious and had greater individual differences. Fucoidan was preferentially enriched in the kidneys, spleen, and liver, with an average residence time of 6.97 h [16].

The results of this study showed that PM was mainly distributed in the kidney and liver, and also in the heart, spleen and lungs after intravenous injection. According to the organizational distribution of PM, we can gain some insights into the pharmacological activity of PM. PM was widely distributed in various organs of the body, which lays the foundation for its various pharmacological activities. Yang et al. found that alginate oligosaccharide increased LDLR expression and intracellular uptake of LDL by hepatocytes in a dose- and time-dependent manner [10]. The high distribution of PM in the liver can support the pharmacological effect of PM and better play the effect of lowering LDL. In addition, the persistent distribution of PM in the spleen supports its immunomodulatory function [7]. The persistent high distribution of PM in kidney suggests that the application of PM in the prevention and treatment of kidney diseases may be explored.

## 4. Materials and Methods

### 4.1. Materials

Kunming male mice (Qingdao Darenfucheng, Qingdao, China); Q-Sepharose, Sephadex G-25, (GE, Boston, WWLP, Boston, MD, USA); TSK-gel G4000 PWxl column (TSK, Fukuroi, Japan); sodium alginate, tyramide, cyanide sodium borohydride, FITC, trichloroacetic acid, cetrimonium bromide (CTAB) (Aladdin, Shanghai, China).

### 4.2. Methods

#### 4.2.1. Preparation of PM

Sodium alginate (100 g) was thoroughly dissolved with pure water (5000 mL), and then concentrated hydrochloric acid was slowly added into the solution to adjust the concentration of hydrochloric acid to 0.5 M. The solution was hydrolyzed in hydrochloric acid at 100 °C for 10 h and centrifuged (5000 rpm, 10 min) to collect the precipitate. Then, the precipitate was dissolved in 8% NaHCO_3_ solution and pH adjusted to 2.86 with HCl, which resulted in a large amount of white flocculent precipitate and yellow supernatant. Discarded the precipitation, and the yellow supernatant was adjusted to pH 7–8 with NaOH solution. Then, added three times volume of 95% ethanol to the yellow supernatant, and white flocculent precipitation was obtained after standing for 6 h. The precipitate was dissolved with 8% NaHCO_3_ solution again, and then pH adjusted to 2.86 with HCl to obtain the supernatant by centrifugation. After processing the supernatant following the above mentioned operation twice, adding excess absolute ethanol to promote dehydration, and removing the alcohol by suction filtration for purification, the crude PM was obtained after drying at 50 °C [17].

The crude PM powder (1.0 g) was dissolved in pure water and filtered with a 0.45 μm membrane filter, and the sample was purified by ion-exchange chromatography (GE, Boston, WWLP, Boston, MD, USA) (Q-Sepharose column, 50 cm × 5 cm, Cl-form). The operation of the AKTA protein purification system (GE, AKTA Explorer10, Boston, WWLP, Boston, MD, USA) was as follows: (1) The mobile phase of pump A was set to ultrapure water, the mobile phase of pump B was set to 2.0 M NaCl, the flow rate was 1.0 mL/min, the upper-pressure limit was 1.0 MPa, and the ultraviolet detection wavelength was 210 nm. (2) After adding the sample to the column to complete the exchange, linear gradient elution was carried out with NaCl at a concentration of 0.5–2.0 M; the main peak elution was collected according to the 210 nm UV absorption curve and phenol sulfuric acid method, and the elution was concentrated and desalted on a Sephadex G-25 column to obtain pure PM.

#### 4.2.2. Purity Analysis and Molecular Weight Determination of PM

A TSK-gel G4000 PWxl column was installed on a high-performance liquid chromatography machine (Agilent 1100, Palo Alto, CA, USA) according to the instructions provided. The flow rate was adjusted to 0.8 mL/min, the UV detector was turned on, column equilibration was performed with 0.15 M NaCl solution for 1 h, and the purified PM and dextran series standards of known molecular weight combined (Mw = 10, 70, 150 kDa). After filtration with a 0.45 μm membrane, the sample was passed through the TSK-gel G4000 PWxl column with the column temperature set to 40 °C and an injection volume of 20 μL. The fractions were eluted with 0.15 M NaCl solution, and a 210 nm UV absorption curve was used to verify the purity and detect the retention time of PM and dextran [18].

#### 4.2.3. Preparation of FITC-PM

PM (2.0 g) was dissolved in 150 mL of 0.2 M phosphate buffer at pH 8. Then, we added 2.0 g of tyramine and 0.75 g of sodium cyanoborohydride, and the mixture was sealed with plastic wrap and kept in a shaker at 37 °C for 4 days. The liquid was centrifuged at 4000 rpm for 10 min to obtain the supernatant. Four times the volume of anhydrous ethanol was added to the supernatant, and the liquid kept at 4 °C for 6 h to obtain the precipitate, which was collected by 4000 rpm centrifugation for 10 min. The precipitate was dissolved in 100 mL of 0.5 mol/L NaHCO_3_, then 10 mg of FITC was added, and the solution was kept at room temperature for 24 h in dark. The AKTA protein purification system (GE, AKTA Explorer10, Boston, MD, USA) was used to pass the sample through a Sephadex G-25 column for chromatography removal of the tyramine, FITC, and salt components, and the purified sample was freeze-dried after FITC-labeling of PM (FITC-PM) [19].

#### 4.2.4. Validation of FITC-PM Labeling

Agar (0.3 g) was dissolved in 30 mL of 10 × TAE buffer and heated in a microwave until it began to boil, three times to dissolve completely. The solution was poured into a gel tank, and a comb inserted immediately before solidification at room temperature. The 10 × TAE buffer solution was added to the gel tank until the liquid level was about 2 mm above the gel surface. FITC (0.5 mg/mL) and FITC-PM (5 mg/mL) solutions were prepared, and an equal volume of glycerol was added. After loading 10 μL of sample into each hole, the voltage was set at 60 V, and electrophoresis was timed for 80 min. The gels were observed with a gel imager (Bio-RAD, Hercules, CA, USA) [12].

#### 4.2.5. Study of FITC-PM Stability

Considering the complexity of in vivo environments, we investigated the stability of FITC-PM at different pH levels (2, 4, 6, 7, 8, 10, 12). The FITC-PM solution was incubated at 37 °C for 24 h, then adjusted to pH 7. Samples were filtered by a 0.45 μm microporous membrane. The stability of FITC-PM was determined by agarose gel electrophoresis [14].

#### 4.2.6. Tissue Distribution of PM in Mice

##### Grouping and Dosing of Mice

Kunming male mice (20–25 g, *n* = 48) were maintained in an air-conditioned room (25 ± 1 °C) with a relative humidity of 50 ± 20% and a 12 h light/dark cycle environment. To facilitate sampling times of 0.5, 1, 2, 4, 8, 12, 24 h, the mice were randomly divided into eight groups. Each test group was injected with FITC-PM solution (dissolved in physiological saline and filtered by a 0.2 μm sterile filter) through the tail vein at a dose of 50 mg/kg at different times, and the injection dose of each mice was 0.1 mL/20 g bodyweight. The control groups were injected with corresponding doses of normal saline [20].

##### Collection and Pretreatment of Blood and Organs

After 0.5, 1, 2, 4, 8, 12, and 24 h of FITC-PM via tail vein injection, mice blood was extracted from the eyeballs, then the mice were sacrificed by cervical dislocation. For blood collection, 4 mL EP tubes were pretreated with 0.2 mL of 4% trisodium citrate solution. The operator grasped the mice’s head to fully expose the eyeball, removed the eyeball with curved-front tweezers, and collected the blood. The blood was centrifuged at 3000 rpm for 15 min to obtain the plasma supernatant.

After the mice were sacrificed by cervical dislocation, the heart, lungs, liver, spleen, and kidneys were collected. The organs were washed three times with prepared normal saline, dried with filter paper, and weighed. We added three times the volume of normal saline to the EP tube containing the liver and added five times the volume of normal saline to the other organs. Finally, the organs were disrupted using a tissue disruptor to obtain tissue homogenate, which was stored at −40 °C.

##### Handling of Organ Homogenates

After adding 100 μL of 10% trichloroacetic acid solution in turn to 200 μL of the plasma and tissue homogenate of each organ, the samples were kept at 4 °C for 30 min, then centrifuged at 10,000 rpm for 12 min to retrieve the supernatant. A 4% CTAB solution (300 μL) was added to the obtained supernatants, incubated at 4 °C for 12 h, and centrifuged at 10,000 rpm for 30 min to retrieve the CTAB-polysaccharide complex precipitate. The precipitate was washed with pure water and then 200 μL of 8% sodium chloride buffer added to dissolve. After pipetting 100 μL of the liquid into a black microtiter plate, a cell-imaging microplate detection system (Bio-Tek, Cytation 5, Norcross, GA, USA) was used to detect the fluorescence intensity (Ex = 485 ± 20 nm, Em = 528 ± 20 nm).

##### Preparation of FITC-PM Standard Curve for the Blood and Organs

We placed 0, 1, 2, 4, 8, 16, 32, 64, and 128 μL of 1 mg/mL FITC-PM solution into separate 1.5 mL EP tubes and added normal saline to a total volume of 150 μL. Then, 50 μL of heart, liver, spleen, lung, kidney tissue homogenate, and plasma were added to each EP tube, resulting in a total volume of 200 μL in each EP tube. Finally, 100 μL of 10% trichloroacetic acid solution was added and kept at 4 °C for 30 min. The solutions were centrifuged at 10,000 rpm for 12 min, and the supernatant retrieved. After adding 300 μL of 4% CTAB solution to the supernatants and incubating at 4 °C for 12 h, they were centrifuged at 10,000 rpm for 30 min to obtain the CTAB-polysaccharide complex precipitate. The precipitate was washed with pure water, and 200 μL of 8% sodium chloride buffer added to dissolve the CTAB-polysaccharide complex. We pipetted 100 μL of the liquid into a black microtiter plate and used a cell-imaging microplate detection system (Bio-Tek, Cytation 5, Norcross, GA, USA) to detect the fluorescence intensity (Ex = 485 ± 20 nm, Em = 528 ± 20 nm).

##### Free-FITC Interference Detection

Using nine 1.5 mL EP tubes, 0, 1, 2, 4, 8, 16, 32, 64, and 128 μL of 0.5 mg/mL FITC standard solution were added, followed by 50 μL of the treated tissue homogenate and normal saline to a total volume of 200 μL. Free-FITC residue was checked according to the method described in Section Preparation of FITC-PM Standard Curve for the Blood and Organs.

#### 4.2.7. Statistical Analysis

Pharmacokinetic data were processed in software PK Solver 2.0 (PKSolver pharmacokinetic pharmacodynamics data processing software V 2.0, Nanjing, China), and the noncompartmental model was used to analyze the pharmacokinetic parameters of this experiment.

## 5. Conclusions

In this paper, we describe our study of the distribution of PM in mice via tail vein injection. The FITC-PM showed high stability in different pH solutions and it could be used for PM distribution and pharmacokinetic studies. We will continue by studying the absorption distribution and pharmacokinetics of FITC-PM after oral administration to gain a more complete understanding of the absorption, distribution, and metabolism of PM in tissues. Through PM absorption mechanism research and application of different methods for studying pharmacokinetic and tissue distribution, PM absorption mechanism, pharmacokinetics, and distribution in the body can be clarified. It is of great significance to improve the efficacy of PM, improve the bioavailability of PM, study the drug delivery pathway of PM, clarify the mechanism of PM pharmacological activity, and develop new pharmacological activity of PM, which can promote the development of PM drugs.

## Figures and Tables

**Table 1:** Main pharmacokinetic parameters of FITC-PM after tail vein administration (50 mg/kg).

Parameter	Unit	Value
K_e_	1/h	0.24
t_1/2_	h	2.85
T_max_	h	0.50
C_max_	μg/mL	235.17
C_0_	μg/mL	389.98
AUC_0–t_	μg/mL·h	567.37
AUC_0–∞_	μg/mL·h	631.48
AUC_0–t/0–∞_		0.90
AUMC_0–∞_	μg/mL·h^2^	1843.15
MRT_0–∞_	h	2.92
Vz	mL	325.46
CL	mL/h	79.18
Vss	mL	231.11

Ke, elimination rate constant; t_1/2_, elimination half-life time; T_max_, time to peak; C_max_, peak concentration; C_0_, initial concentration; AUC, area under the curve; AUMC, area under the curve by statistical moment; MRT, mean residence time; Vz, volume of distribution; CL, clearance rate; Vss, steady-state distribution volume of intravenous infusion.
